# Validation of Marker-Less System for the Assessment of Upper Joints Reaction Forces in Exoskeleton Users

**DOI:** 10.3390/s20143899

**Published:** 2020-07-13

**Authors:** Simone Pasinetti, Cristina Nuzzi, Nicola Covre, Alessandro Luchetti, Luca Maule, Mauro Serpelloni, Matteo Lancini

**Affiliations:** 1 Department of Mechanical and Industrial Engineering (DIMI), University of Brescia, 25123 Brescia, Italy; simone.pasinetti@unibs.it (S.P.); matteo.lancini@unibs.it (M.L.); 2 Department of Industrial Engineering (DII), University of Trento, 38123 Trento, Italy; nicola.covre@unitn.it (N.C.); alessandro.luchetti@unitn.it (A.L.); luca.maule@unitn.it (L.M.); 3 Department of Information Engineering (DII), University of Brescia, 25123 Brescia, Italy; mauro.serpelloni@unibs.it

**Keywords:** gait analysis, multiple Kinects, calibration, body kinematic, shape and motion analysis, sensor fusion, measurements

## Abstract

This paper presents the validation of a marker-less motion capture system used to evaluate the upper limb stress of subjects using exoskeletons for locomotion. The system fuses the human skeletonization provided by commercial 3D cameras with forces exchanged by the user to the ground through upper limbs utilizing instrumented crutches. The aim is to provide a low cost, accurate, and reliable technology useful to provide the trainer a quantitative evaluation of the impact of assisted gait on the subject without the need to use an instrumented gait lab. The reaction forces at the upper limbs’ joints are measured to provide a validation focused on clinically relevant quantities for this application. The system was used simultaneously with a reference motion capture system inside a clinical gait analysis lab. An expert user performed 20 walking tests using instrumented crutches and force platforms inside the observed volume. The mechanical model was applied to data from the system and the reference motion capture, and numerical simulations were performed to assess the internal joint reaction of the subject’s upper limbs. A comparison between the two results shows a root mean square error of less than 2% of the subject’s body weight.

## 1. Introduction

About 90,000 persons each year are made dependent on a wheelchair for mobility due to a Spinal Cord Injury (SCI) [1]. In recent years, wearable robots were developed to make it possible for people with SCI to walk. As proven by scientific studies [2], these solutions have been successful in reducing the development of secondary diseases such as obesity, diabetes, sores, and osteoporosis. In improving the quality and duration of life of people with SCI, and, at the same time, lead to the overall containment of the costs of the health system.

In previous research activities, our research group worked on the experimentation of a commercial exoskeleton, which is a *REwalk* personal version (P5), with SCI subjects. The present paper is heavily based on these previous works and it is meant to expand the research further. The experimentation took place in facilities dedicated to the recovery of paraplegic subjects. The subjects were trained to use the exoskeleton in gait labs with the support of specialized physiotherapists, acting both as a caregiver and instructor to correct the posture during the walk session. As described in Reference [3], the subjects, after wearing the exoskeleton, were equipped with a pair of crutches that (i) served as an aid to walking along a predefined path and (ii) were instrumented with strain gauge bridges and a triaxial accelerometer to measure both axial and shear forces, tilt angles, and impact time on the ground in real-time [4,5,6]. A simplified biomechanical model, based on the *OpenSim* platform [7], was designed to represent the system formed by the subject, the exoskeleton, and the crutches. These previous works aimed at providing a quantitative measure of the internal forces acting on the shoulders of the patient to improve the training performance and avoid incorrect use of the exoskeleton that could lead to pathologies at the shoulders. The results of the work were encouraging. The accuracy of the measurement of the internal forces was proven adequate to enhance the therapist’s evaluation and the patient’s engagement.

In this paper, we want to expand the research further. Monitoring upper joint reaction forces and gait objectively is only the first step for the correct, continuous, and autonomous practice of the subject in living environments. One of the issues is represented by the fact that the acquisition of the joint trajectories is carried out using marker-based optical tracking systems (MBS), which are the gold standard for clinical gait assessment [8,9]. The research in this field has produced several MBS that are currently market available. Among them, the *Vicon* (by Vicon Motion Systems Ltd, Oxford, OX5 1QU, UK), the *Xsens* (by Xsens Technologies, Enschede, PR, Netherlands), the *Phase Space* (by PhaseSpace Inc., San Leandro, CA, USA), the *Optitrack* (by NaturalPoint Inc., Corvallis, OR, USA) and the *Smart-DX* (by BTS Bioengineering, Garbagnate, MI, Italy) are marker-based optical systems that provide very accurate human capture. However, they suffer from limitations. In particular, the high cost and computational complexity limit their adoption to the gait labs [10]. Even for those who can have access to the gait labs, the setting available in the laboratory is not representative of realistic living environments, either for the presence of the clinical staff, for the limited training time slots, and for the typologies of paths that can be reproduced in indoor clinical settings.

Wearable IMU sensors are being intensively studied for real-world applications and could represent a reasonable choice for extending the exoskeleton-based training in living environments. They are used to estimate the orientation of the human body in healthcare-related applications, such as fall detection of elderly people [11,12], body and postural orientation [13,14], sports and athlete’s limb dynamics [15,16,17], gaming [18], and robotic prosthetic body parts especially upper limb rehabilitation [19,20]. Gait analysis is also successfully carried out using wearable IMUs, such as in References [21,22].

Their use for human motion capture presents the advantages of ubiquitous performance, high accuracy, small size, low cost, and reduced complexity and costs. However, their adoption is not free from limitations. Combining body acceleration and orientation changes in accurate measurements is still an issue since the accelerometers measure both linear and gravitational accelerations. Drift compensation requires fusing different sensors technologies, as in References [23,24,25] or developing sophisticated elaboration algorithms [26]. The presence of the exoskeleton is another issue because it affects the quality of motion recognition. To overcome these limitations, different solutions have been proposed in the literature, such as the use of neural networks-based analysis [27], sensor data fusion to estimate the forces exerted by the foot on the sole [28], and in-shoe pressure mapping to identify the gait phases [29]. However, increasing the number of sensors means increasing the whole system complexity both in terms of system set-up and of data analysis, and does not help its usage in uncontrolled environments. A different alternative could be to directly acquire the signal generated by the exoskeleton joint angular sensors, as in Reference [30], but this solution does not apply to commercial exoskeletons, which usually are completely closed systems.

The examination of the state-of-the-art system has convinced us that these sensors need contact with the exoskeleton (lower limbs) and the body segments of the subject (upper limbs) constitute a significant obstacle to their continuous use since it requires time and precision in positioning. For example, marker setting is time-consuming and small errors in the positioning of either MBS markers or IMUs, as well as soft tissue artifacts, induce large errors in estimating the joint centers [31]. In addition, a significant degree of effort of the subject is necessary. This aspect should not be underestimated because the subject could be unable to collaborate or not compliant to collaborate.

Vision-based marker-less systems (MLS) represent a valuable alternative to the above-mentioned systems. Both 2D and 3D cameras can be used to acquire the RGB image or the depth information of the subject and suitable procedures and methods have been developed to track the patient’s motion. MLS can provide a reasonable option to develop a motion capture system suitable for functional assessment activities in living environments, provided that the adopted devices guarantee good accuracy of the estimate of both the upper limb forces and the gait quality [32,33]. At present, among the commercial devices suitable for the application, the *Microsoft Kinect^TM^* sensor deserve particular attention due to the cost-effectiveness and portability of this device [34,35]. This also provides the subject’s skeleton estimation based on 25 joints of the human body [36]. This embedded capability has captured the interest of many research communities in a large number of fields such as multimedia [37], gaming [38], robotics [39], gesture recognition [40,41], and motion analysis [42,43,44]. At present, the Kinect is the most investigated sensor for advanced and affordable applications in the healthcare field such as elderly care and rehabilitation [45,46,47,48,49], functional assessment activities and posture [50,51,52,53], and daily life activities [54]. The accuracy and reliability of the Kinect sensor in gait assessment and motor function has been extensively investigated. Works published in the last five years provide comparative analyses of multi-Kinect systems benchmarked against MBS setups used as the gold standard, and all show good-to-excellent agreement with the gold standard in gait assessment [55,56,57] and motor function [58,59].

In this paper, a multi-Kinect system is proposed for providing the joint trajectories of both upper limb body segments and exoskeleton lower limbs. The devices’ position and the orientation have been optimized to maximize the coverage of the training path, which minimizes the occlusions of body/exoskeleton segments. Suitable multi-view calibration, previously developed by some of the authors, has been used to perform the extrinsic calibration of the Kinects, i.e., to estimate the roto-translation matrices that map the skeletons produced by every single Kinect in a common reference system [60]. To comply with the fact that the Kinect-embedded skeletonization is designed to optimally track the front side of the body while non-frontal views show larger errors [61], a suitably developed Kalman-based algorithm is proposed to fuse every single skeleton in a single one, which represents the joint trajectories in the global reference system. The system is benchmarked against the BTS system, used as the gold standard. To this aim, the experimental work presented in this scenario has been carried out using the same setup as the one in Reference [3] where the subject walks in the gait lab, and is simultaneously tracked by the multi-Kinect marker-less (ML) system and by the BTS marker-based (MB) system.

While assessing the impact of external forces on joints is important to prevent injuries, a direct measurement is not feasible. Hence, a field of biomechanical models are being developed to address different issues as well as software solutions to handle this issue. The implementations of these models feed on motion capture and on external forces measurements to assess the moments and forces acting internally. Complex models are not always the best solution since their validation could be difficult [62], and the trade-off between computation-time and results could be complex [63]. In our case, the acquired data feed a simplified mechanical model developed in OpenSim [64] that, using the information from both the instrumented crutches and the force platform, estimates the internal forces exerted at the shoulders. Since this model was used in a previous assessment of the same activity [3], it was used as a reference process for the acquired data.

## 2. ML Multi-Kinect Motion Tracking System

**Figure 1:**
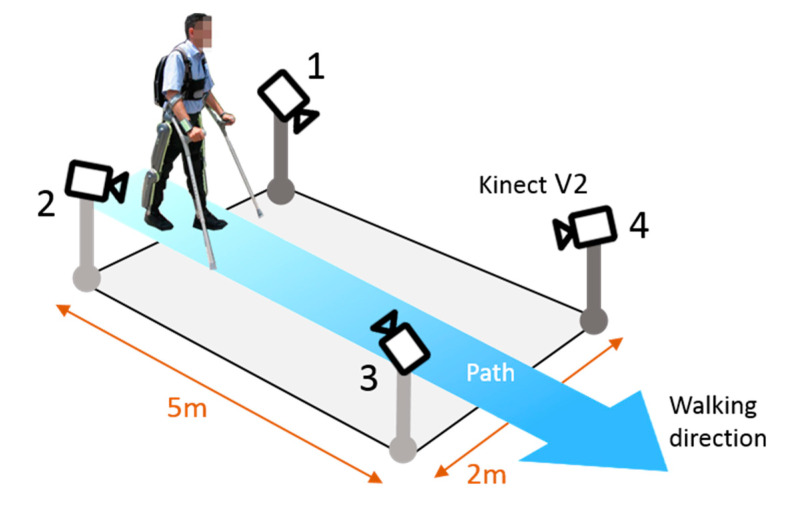
Orientation of the 4 Kinects in the measurement area, and direction of the walking.

The ML multi-Kinect system is composed of four Kinects V2, which provide both an RGB sensor (1920 × 1080 pixels) and a Time of Flight (ToF) depth sensor (512 × 424 pixels) spanning a range of 0.5 m–5 m and 70° × 60° field of view at a maximum frame rate of 30 fps and maximum standard uncertainty of 18 mm at 5 m [35]. The Kinects are organized in the measurement space as schematically shown in Figure 1. Their mutual distance along the direction of walking is 5 m, and the perpendicular distance is 2 m.

Due to the high amount of data sent by the Kinect sensor, it requires a dedicated USB 3.0 bus and external 12 V power. The Microsoft APIs developed for the Kinect V2 offer the skeletonization functionalities, which are strategic in our application for assessing the human kinematics. However, they are not compliant with the use of multiple Kinects on a single PC. For this reason, in our system, each sensor is connected to a dedicated client unit, an Intel NUC PCs, which provides RGB images, depth images, and the skeleton of the subject. The clients are organized in a local LAN network mastered by a server unit. The clients communicate with the server using an MQTT protocol. The server acquires the skeletons from every single client.

**Figure 2:**
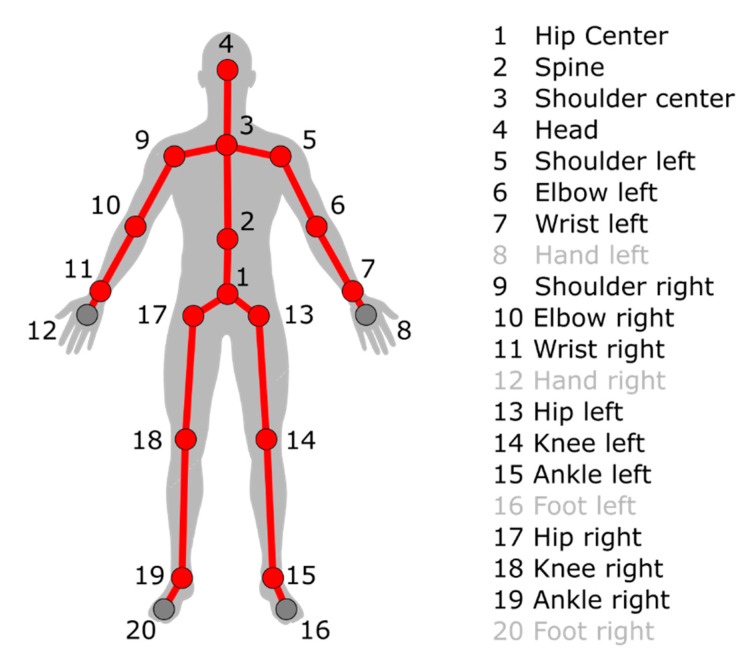
Skeletal data provided by the Kinect V2. On the left is the graphical representation. The node numbering is on the right. The 16 nodes considered in our algorithm are the ones in red.

Figure 2 shows the skeletal data provided by each Kinect. In our implementation, nodes 19 and 15 collapse with nodes 20 and 16, respectively, and nodes 11 and 7 collapse with nodes 12 and 8, which resulted in a total of 16 nodes. This choice was motivated by the need to account for the most stable nodes, i.e., those measured with acceptable precision (5 cm, as stated by Reference [59]), and by the fact that the hand is, in our test case, rigidly linked with the lower arm, since they are both strapped to the crutch, which constitutes a single rigid body (lower arm, hand, and crutch).

The server unit implements the three following tasks: (i) skeleton synchronization, (ii) skeleton registration, and (iii) skeleton fusion. Skeleton synchronization and skeleton registration have been previously developed by some of the authors and are exhaustively described in Reference [60], while skeleton fusion has been developed specifically for the application of interest and is detailed in Appendix A.

**Skeleton synchronization:** when dealing with multi-Kinect systems, skeleton synchronization is mandatory to avoid possible misalignments among the skeletons captured by each device (client). Even if the acquisition frequency is very stable, macro relative time delays greater than the sampling period (∆t = 33 ms) are possible and result in noticeable misalignments among the captured skeletons, which, in turn, hinder their accurate registration in a common reference. To prevent this situation, in Reference [60], a dedicated synchronization software has been implemented on the multi-Kinect network, based on the *Network Protocol Time* (NTP) by Meinberg. From experimental tests, this manages to provide a temporal synchronization among the clients with an expanded uncertainty of ±5 ms (P = 95%).

**Skeleton registration:** as described in Reference [60], this is accomplished by a dedicated calibration framework, aimed at estimating the extrinsic parameters. These are the rotation matrix and the translation vector mapping the skeleton node positions from the coordinate system local to each Kinect to the arbitrarily chosen global measurement system. In Reference [60], the authors focused on the development of a simple, fast, and easily reproducible calibration procedure, based on the use of a colored ball as the calibration tool, which can be moved by hand in the Field of View (FoV). Unlike the checkerboards commonly used to calibrate multiple Kinects [42], the calibration tool is lightweight, easy to handle, and suitable for calibration in non-technical environments. The method is based on the acquisition of the RGB and depth images of the target by each local client and on the time synchronization and spatial matching of the 3D trajectories of the calibration tool. In their experiments, a standard uncertainty of the extrinsic parameters was assessed to be lower than 2 mm and 10^−2^ radians, which is better than the uncertainty values attainable using state-of-the-art, bulky calibration checkerboards [60].

**Figure 3:**
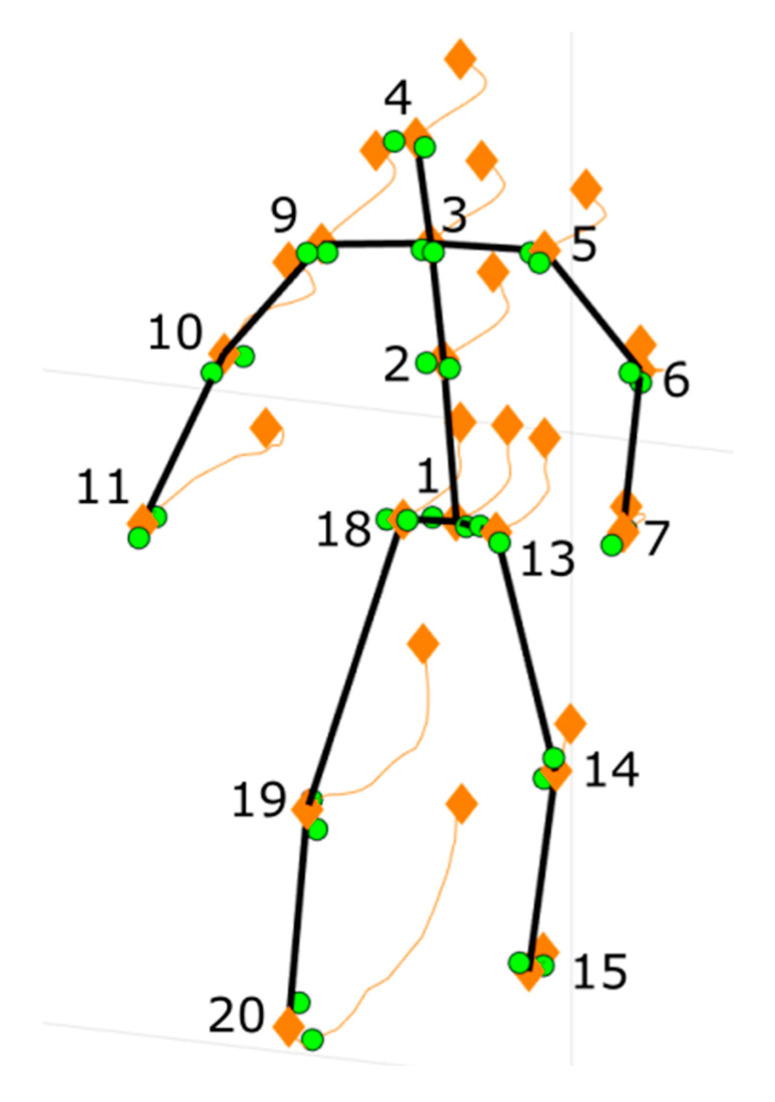
Output of the fused skeleton and the trajectories of the nodes in time. Green dots represent the nodes measured from different Kinects. Orange diamonds indicate the nodes of the fused skeleton and orange lines are their trajectories.

**Skeleton fusion:** this procedure was specifically designed for this work and is described in detail in Appendix A. It is carried out using a probabilistic filtering approach. This choice was motivated by the fact that the estimated node position varies discontinuously and is noisy due to occlusions, to the influence of non-frontal views, and to the uncertainty inherently associated to both the measured nodes and the calibration parameters. Among probabilistic filtering methods, the Kalman filter has been chosen [65,66,67]. An example of the fusion process is shown in Figure 3. In this case, green dots represent measurements $y_{n}^{m}$ for m = [1, 2] and the nodes for the considered skeleton model at time k. Orange diamonds correspond to $X_{p}^{n}$ at the time instant 0 and k, respectively. Orange lines are the node trajectories from time 0 to time k.

## 3. Measurements Set-Up

### 3.1. Hardware

**Figure 4:**
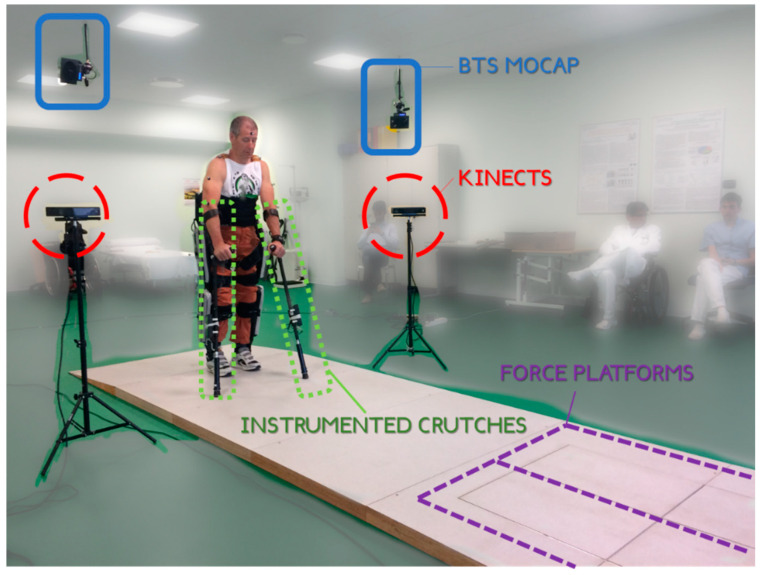
The gait lab used to perform the validation. The instrumentation used is highlighted.

The measurement set-up used to validate the ML multi-Kinect system is shown in the photograph of the gait lab taken before starting the trials (Figure 4). The four Kinects (red round frames) have been positioned following the geometry presented in Figure 1. The gait lab was equipped with the Smart-DX system for motion capture and with four P-6000 load platforms. The Smart-DX system is based on eight wall-mounted infrared video cameras (four cameras are visible in the photograph, framed in the blue rectangles) with a sampling frequency of 2 kHz, a resolution of 4 Mpx, and a standard accuracy of 0.1 mm for the static position for the whole walking range of 6 m. The P-6000 load platforms (framed in the purple rectangle) provide a 6 N overall expanded uncertainty (P = 99%) on each axis. They were split in two separate rows, hidden in the floor, to measure the left and right foot separately. Ground reaction forces at the feet and gait events were captured synchronously with the data set provided by the Smart-DX system.

The crutches, framed by the green rectangles in the figure, were instrumented using a set of four 350 Ω strain gauges applied to the external surface of each crutch, in a full bridge configuration, to detect axial force. One *Arduino Nano* V3.0 board acquires force data as well as accelerations of each crutch thanks to an IMU (*LSM9DS1* by STMicroelectronics, 1204 Genève, Switzerland, $\pm 20\;m/s^{2}\;\pm \;4\;rad/s\;$). In this application, we chose to acquire the voltage signal from the bridge with an ADC channel. Therefore, the resolution is 10 bit giving a voltage of about 4.8 mV. These values are applied frequently for similar applications. For example, in Reference [68], an Arduino board is used. The crutches used in the experimentation were improved with respect to those described in References [1,3]. They have a simplified electronic circuit permitting an easier and prolonged functioning and improved accuracy thanks to the compensation of bridge hysteresis and non-linearity.

All data were transmitted in real-time using a Bluetooth module (*ESD200*) with a sampling frequency of 40 Hz and recorded using a custom-made software developed in *LabVIEW* and running on a Bluetooth-enabled personal computer. Each crutch was calibrated to its full range of 600 N, and evaluated by comparison with industrial precision load cells, which show an expanded uncertainty (1-h drift, hysteresis, non-linearity P = 99%) of 6 N, and a resolution of 1 N. The crutches and their validation are deeply described in Reference [11].

Before the measurement campaign, the calibration of both crutches was verified using an industrial load cell (*Gefran TH*) and applying a periodic loading. The same test was performed at the end of the test campaign to ensure that usage and transportation did not alter the static sensitivity of the system. The crutches’ length is adjustable and was extended to adapt the crutches to the user’s preference. Before each test, the crutches were lifted from the ground and any residual offset in the force reading was compensated.

### 3.2. Software

A multibody numerical simulator is used to assess the internal reaction from kinematics and dynamics using a biomechanical model. The biomechanical model has been implemented using the open source OpenSim software developed at the Stanford University [7,64].

**Figure 5:**
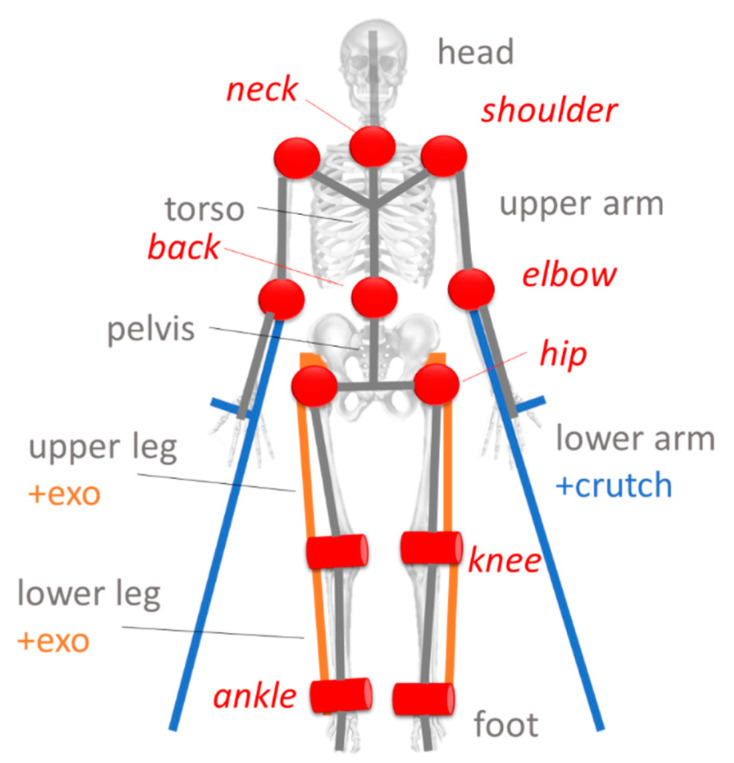
Mechanical model used to interpret the kinematic and dynamic data. Body segments are shown in black. Joints are shown in red. Cylinders indicate cylindrical joints. Balls indicate spherical joints.

The patient is modeled as a passive multibody system reproducing a simplified version of the skeletal portion of the human body. Following the guidelines provided in Reference [62], both muscular components and the soft tissues are neglected to minimize the number of the model parameters. The model chosen was detailed in Reference [5]. As shown in Figure 5, 12 joints (four cylindrical and eight spherical) and 13 rigid bodies are considered (the crutches are rigidly connected to the forearms), which results in 31 rotational degrees of freedom. Inertial properties, mass, and dimension of each segment are based on the anthropometric data taken from Reference [69]. In addition, the length of each body segment is corrected using the kinematic data provided by the vision system (either marker-based or marker-less) [70].

The influence of the exoskeleton segments and components on the corresponding body segment has been accounted for by adding the following masses: 5 kg to each lower leg and 5 kg to each upper leg as well as 3 kg to the torso section (motor and batteries). The influence of the crutches has been modeled by adding 1.5 kg to each forearm. In addition, the center of mass and the inertial properties of the forearms have been computed by simulating numerically the system formed by the forearm, the hand, and the crutch using suitable computer assisted design software (*Solidworks*, by Dassault Systèmes, Waltham, MA, USA). Upper and lower leg centers of mass have not been corrected. The model formulation clearly hinders from evaluating the interactions between the patient and the exoskeleton such as those induced by muscle contractions, muscle stiffness, and spasticity as well as the influence of the relative movements between the body and the exoskeleton segments. However, this formulation is very simple, and represents a reasonable compromise between computing efficiency and data accuracy.

### 3.3. Protocol

The experimentation has been performed on a single user of a P5 Rewalk exoskeleton, who was considered an expert user, with more than 10 h per week for at least nine months of domestic usage. In Figure 4, the patient is visible at the start position of the test path. The subject was a 58-year-old male (68 kg, 1.78 m), right-hand dominant, with a complete spinal cord injury at level T12. The preparation of the subject was necessary in view of the acquisition of the kinematic data from the marker-based motion capture.

**Figure 6:**
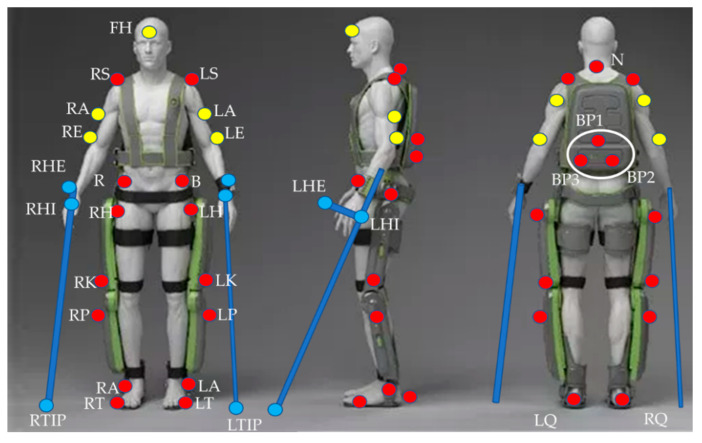
Position of the retroreflective markers. Red dots indicate markers from the original Davis protocol, yellow dots indicate added markers on the subject, and blue dots indicate markers placed on the crutches.

Twenty-five retro-reflective spherical markers (20 mm in diameter) were applied on both the patient and the exoskeleton by following a Davis protocol [71], which have been suitably modified to account for the exoskeleton [72,73]. Hence, this obtains a custom protocol purposely designed for this case. These are the markers represented by red dots in Figure 6. With respect to the standard Davis protocol, markers RK, RP, LK, and LK were positioned about 10 cm laterally with respect to their original position. In addition, the sacral marker was replaced by three markers in triangular configuration placed on the exoskeleton’s battery pack. Moreover, to get a complete assessment of each degree of freedom of the model, five markers (yellow dots in the figure) were added in correspondence to the subject forehead, 2 cm higher than the nose (FH) on each elbow (RE, LE) and on the upper arm at the midpoint between the elbow and shoulder (RA, LA). To account for the crutches, six further markers (blue dots) were positioned on the crutches tip (RTIP, LTIP), on the handle end (RHE, LHE), and at the intersection between the handle and the crutch (RHI, LHI).

The subject received no instruction on his movement and was free of walking along the path on his own accord. He reported no difficulty using the device and showed no sign of fatigue during or after the tests. A total of 20 walking tests were performed in both directions. During the tests, the ML multi-Kinect system, the Smart-DX MB system, the load platforms, and the crutches simultaneously acquired the respective data. The skeleton fusion was carried out by following the procedures in Appendix A and the tracking of the physical markers was carried out by following the procedures embedded in the BTS device. The following four data sets were acquired in parallel.

1. The vector $x_{P}^{n}$ of the trajectories of the N *nodes* representing the fused skeleton of the subject, provided by our ML multi-Kinect system,
2. the vector $x_{P-Markers}$ of the trajectories calculated by the BTS Smart-DX vision system of the *Physical Markers* shown in Figure 6;
3. the GF_RF_ data set of the measurements provided by the force platforms of both the forces exchanged between the subject feet, the ground, and the gait phases, and
4. the GC_RF_ data set of the measurements from the crutches of the forces exchanged between the upper limbs of the subject and the ground. In addition to the forces, the impact times of the crutches with the ground are acquired.

## 4. Validation Methodology

The methodology used to assess the ability of the ML multi-Kinect motion capture system to estimate the internal forces acting at the patient upper limbs is based on the following steps.

- **Kinematic data set alignment**: since the ML multi-Kinect system and the MB BTS System are not synchronized to each other, the skeleton trajectories of vector $x_{P}^{n}$ must be aligned in time with the trajectories of vector $x_{P-Markers}$. This task is carried out by the procedure presented in Section 4.1.
- **Mapping skeleton nodes to virtual markers**: the skeletal data $x_{P}^{n}$ provide the trajectories of the joints of the body-exoskeleton combination while the BTS data $x_{P-Markers}$ refer to the trajectories of the physical markers placed on the segments of the body-exoskeleton combination. This last set is the one expressed in the correct form and then elaborated on by the biomechanical model. Starting from the joints trajectories, it is mandatory to calculate the trajectories of new points (thereafter called virtual markers), placed where physical markers would be. A suitable procedure presented in Section 4.2. has been designed to perform this task. The output is represented by the data set denoted by $x_{V-Markers}$.
- **Inverse Dynamic Analysis**: the core of the process is to solve the set of dynamic equilibrium equations that represent the biomechanical model of the patient body. This model performs the inverse kinematics and the inverse dynamic analysis of the gait, which provides the indirect estimate of the subject internal forces at the upper limbs. The analysis based on the biomechanical model is run twice. The first run is carried out using as input data sets $x_{P-Markers}$, GF_RF_, and GG_RF_. The output is used as the reference measurement of the internal forces at the upper limbs. The second run uses as inputs data sets $x_{V-Markers}$, GF_RF_, and GC_RF_. The output of this run represents the estimate of the internal reaction forces at the upper limbs measured by the ML system.
- **Validation of the M-K marker-less vision system:** it is a comparison of those estimated with the reference upper limbs’ internal reaction forces. The quantities evaluated for this comparison were the longitudinal, lateral, and vertical components of the internal joint reactions in the right and left shoulders and elbows for a total of 12 forces. To provide an overview of the validation, the root mean square of the difference between reference and estimated values of these 12 forces was computed.

### 4.1. Kinematic Dataset Alignment

The alignment of data set $x_{P}^{n}$ to data set $x_{P-Markers}$ has been achieved by measuring the following.

- The distance between the right and the left knee: $D1_{Kinect}$ and $D1_{BTS}$ for the M-K marker-less system and the BTS system respectively,
- the distance between the right knee and its contralateral elbow: $D2_{Kinect}$ and $D2_{BTS}$,
- the distance D3 between the left knee and its contralateral elbow: $D3_{Kinect}$ and $D3_{BTS}$.

These distances have been derived using the position of the corresponding nodes of the fused skeleton and the position of the corresponding physical markers of the BTS model (Table 1).

The idea is that the patient motion represents an invariant during the walk. Hence, the evolution of the corresponding distances measured by the two systems shows the same periodicity in time. This was used to align the M-K marker-less system to the reference BTS. The correlation function between values was computed, and the delay corresponding to its maximum value was used as a time offset between the systems.

**Figure 7:**
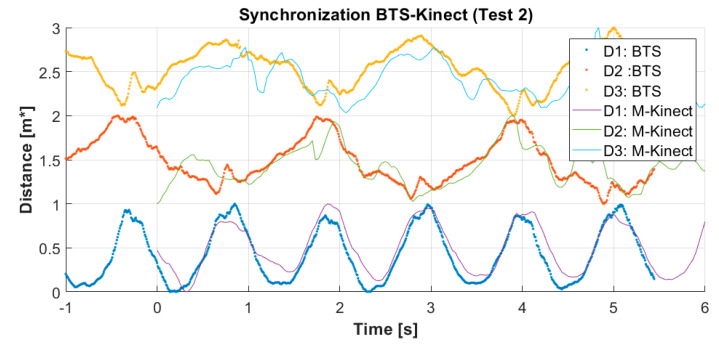
Example of distances D1, D2, and D3 measured using the datasets in Table 1 as a function of time, after alignment using correlation. Dots indicate the reference (BTS) system, which lines the M-Kinect system.

A decimation was then used to sub-sample all collected data to 30 Hz. As an example, the behavior of D1, D2, and D3 measured using the $x_{nodes}\;$ and the $x_{P-Markers}$ data sets, respectively, displayed as a function of time is shown in Figure 7. The periodicity is evident especially for the plots of values D1.

### 4.2. Mapping Nodes to Virtual Markers

This procedure is based on two steps: the first is to obtain body segments (head, limbs, torso, pelvis, and feet) from skeleton nodes. The second aims at estimating the position of virtual markers placed on each segment.

#### 4.2.1. Kinematic Model Conversion

The conversion of the skeleton nodes in body segments of the reference model is performed by defining a local reference system for each segment, as follows.

**Figure 8:**
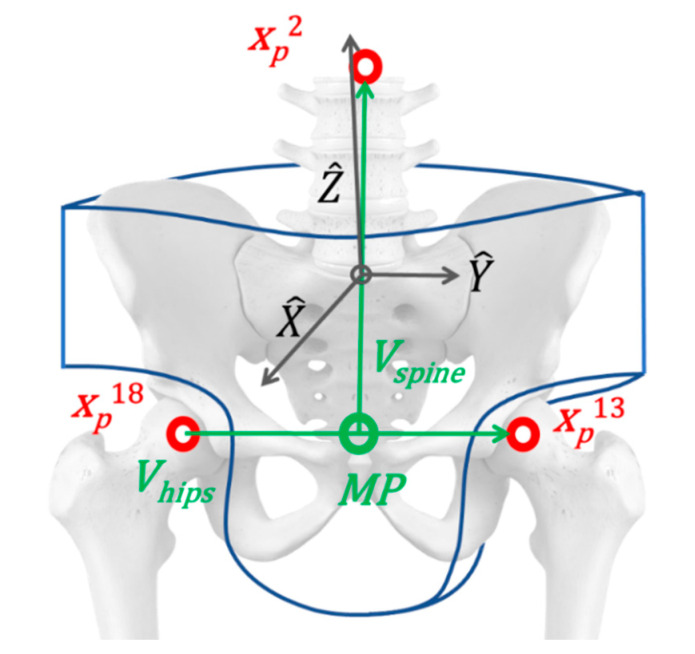
Kinematic conversion of the pelvis segment (in blue). The nodes from the M-K system are in red. The computed vectors and points are in green. The segment’s local reference system are in black.

- **Pelvis:** the pelvis structure is built starting from the hip nodes $x_{p}^{18},\;x_{p}^{13}$. The vector $V_{hips}$ that links nodes $x_{p}^{18},\;x_{p}^{13}$ is set as the $\hat{Y}$ principal direction for the local reference system. Its midpoint MP is used as a base to compute the spinal vector $V_{spine}$ by using $x_{p}^{2}$ as a reference endpoint node located in the middle of the torso. $V_{spine}$ is used as the temporary ${\hat{Z}}^{*}$ principal direction, (temporary because $V_{hips}$ and $V_{spine}$ are usually not orthogonal one to the other). Vectors $V_{hips}$ and $V_{spine}$ are normalized (${\hat{V}}_{hips}$ and ${\hat{V}}_{spine}$) and used to compute the $\hat{X}$ vector, which points to the frontal direction. Lastly, the cross product of $\hat{X}$ and $\hat{Y}$ provides the orthogonal vector $\hat{Z},$ which completes the base for the pelvis reference system $H_{pelvis}.$ The origin of the local reference system is defined by moving the hips midpoint MP along the vector ${\hat{V}}_{spine}$ of a fixed quantity $\delta_{spine}.$ Equation (1) reports the full notation, and Figure 8 shows a schematic representation of the geometry.
  (1) $$T_{H_{pelvis}}=MP+\delta_{spine}\cdot {\hat{V}}_{spine}\;\;\;\;R_{H_{pelvis}}=\left[\hat{X}=\frac{\hat{Y}\times {\hat{Z}}^{*}}{\left|\hat{Y}\times {\hat{Z}}^{*}\right|},\hat{Y},\;\hat{Z}=\frac{\hat{X}\times \hat{Y}}{\left|\hat{X}\times \hat{Y}\right|}\right]\;\;\;\;\;\;\;H_{H_{pelvis}}=\left[\begin{array}{ll} R_{H_{p}} & T_{H_{p}}\\ 0 & 1 \end{array}\right]\;\;\;$$
- **Torso:** for the torso, similarly to the pelvis, the initial reference vector is computed from the nodes of the shoulders. The spinal vector computed for the pelvis is used in a vertical direction. The same normalization procedure is applied.
- **Limbs:** the reference systems are settled in correspondence with the nodes, from the inner to the outer part of the body. For the upper arm is the shoulder $P_{shoulder},$ while, for the lower arm, the elbow $P_{elbow}\;\mathrm{is}\;\mathrm{used}.$ The wrist $P_{wrist}$ and the heel represent the endpoints of the respective limbs. $\hat{X}$ is aligned with the joint while $\hat{Y}$ is computed as the normal vector of the plane defined by the motion of the considered limbs frame-by-frame. $\hat{Z}$ is derived from the cross product of the previous two. Equation (2) reports the calculation for one of the upper limbs.
  (2) $${\hat{Y}}_{l}=\frac{\left(P_{e}-P_{s}\right)\times \left(P_{w}-P_{s}\right)}{\left|\left(P_{e}-P_{s}\right)\times \left(P_{w}-P_{s}\right)\right|}$$
- **Foot:** the foot is defined starting from the definition of the ankle $H_{ankle}.$ The same orientation is applied to two virtual points.
  (3) $$H_{foot_{1}}=H_{ankle}\cdot \left[\begin{array}{llll} \alpha_{foot} & 0 & \gamma_{foot} & 1 \end{array}\right]{}^{\prime }\;\;\;\;H_{foot_{2}}=H_{ankle}\cdot \left[\begin{array}{llll} \beta_{foot} & 0 & \gamma_{foot} & 1 \end{array}\right]{}^{\prime }$$
- **Crutch:** since the patient’s forearms are strapped to the crutches, each forearm-crutch couple is considered a single rigid body, including the hand. Two virtual points are defined from the reference system of the wrist $H_{wrist}$ to model the crutch. Given its geometry, a point is defined for the handle and one is defined for the endpoint.
  (4) $$H_{crutch_{h}}=H_{wrist}\cdot \left[\begin{array}{llll} 0.1 & 0.1 & 0 & 1 \end{array}\right]{}^{\prime }\;\;\;\;H_{cruch_{ep}}=H_{wrist}\cdot \left[\begin{array}{llll} 0.5 & 0 & 0 & 1 \end{array}\right]{}^{\prime }$$

All the numeric values $\delta_{spine},$ $\alpha_{foot},$ $\beta_{foot},\;\mathrm{and}$ $\gamma_{foot}$ were defined by considering the anthropometric tables [69] scaled to the subject [70].

#### 4.2.2. Virtual Markers Definition

The reference system (by BTS) records the trajectories of the markers. To make sure that both systems were handled by following the same numerical process while simulating the joint reaction, virtual markers were created for the kinetic data measured using the Kinect system. Each segment in the model is considered rigid and each marker trajectory $x_{v-marker}$ could be obtained by multiplying the relative position of the marker in the body segment $P_{v-marker},$ which is constant in time with the reference system of the body segment $H_{segment},$ which is time-dependent, as shown in Equation (5).

(5) $$x_{v-marker}=H_{segment}\cdot P_{v-marker}$$

The relative positions $P_{v-marker}$ of the markers with respect to their parent bodies are input parameters for the OpenSim model. An incorrect definition of these parameters would lead to an inaccurate kinematic analysis [61], which, in turn, would lead to an incorrect comparison between the two systems. To avoid this, the relative position $P_{v-marker}\;$of the marker with respect to the reference system was assessed for both systems, starting from the marker trajectories recorded by the reference marker-based system, $x_{p-\mathrm{marker}},$ and the bodies’ reference systems measured by the marker-less motion capture system, $H_{\mathrm{segment}},$ as shown in Equation (6).

(6) $$P_{v-marker}=H_{\mathrm{segment}}^{-1}\cdot x_{p-\mathrm{marker}}$$

**Figure 9:**
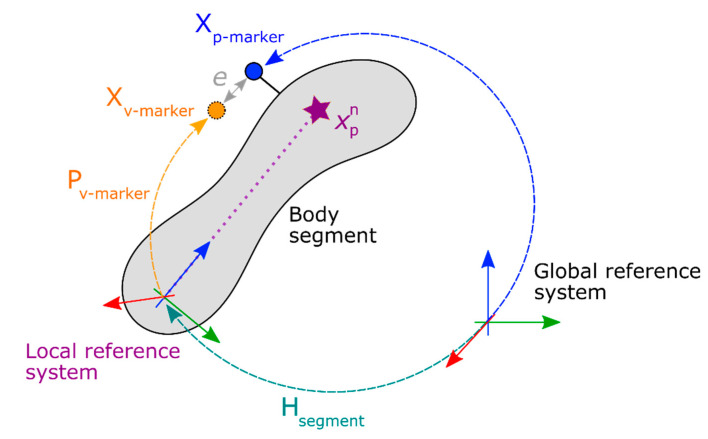
Definition of the virtual markers’ position based on the real marker relative position and the local reference system of the body measured using the multi-Kinect system.

For each test, an optimization system (genetic algorithm ‘*ga*’ in *Matlab*) solved Equation (6), which minimized the distance $e_{marker}=\|x_{p-\mathrm{marker}}-x_{v-\mathrm{marker}}\|$ between the trajectories of the Kinect virtual marker and its associated BTS marker. To consider the relative rotation between the two systems references, a global roto-translation matrix was included in the guesses on the numerical optimization. Using the transformation matrices, the delay and the virtual markers‘ trajectories, it was possible to represent the skeleton from the marker-less system in the same reference system (BTS-based), which mimicks marker-based measurement results. Figure 9 shows the graphical definition of the virtual markers’ positions.

### 4.3. Inverse Dynamic Analysis

After having scaled the mechanical model to the patient characteristics and to the exoskeleton mass, as reported in Section 3.2, the OpenSim numerical simulator was used to perform two sequential analysis.

1. the inverse kinematic analysis, which provided the degrees of freedom (DoF) value of the model in time, starting from the markers (either virtual or physical) positions,
2. the inverse dynamic analysis, to compute the motor torques applied to joints, and the internal joint reaction forces, starting from the kinematic results and the external forces acting on the body (GC_RF_ data and GF_RF_ data from the instrumented crutches and the force platforms, respectively).

The result of those analyses, which were used for validating the system due to their clinical significance, are the joints’ internal reaction forces related to the upper limbs, listed in Table 2. While for kinematic analysis, the whole 6-m walk was used. Only the two central steps of each walk were used for the dynamic analysis since the force platforms could accommodate only two steps (see Figure 1 and Figure 4).

To provide data easily comparable with medical literature, two different normalizations were performed, following standard clinical practice [74].

- **Gait phase normalization:** ground reaction forces were used to detect the gait events (heel contact, toe off), and time was scaled to be 0% at the first right heel contact and 100% at the second right heel contact on the force platform.
- **Body weight normalization:** results in term of force were divided by the weight of the system composed by the subject, the exoskeleton, and the crutches.

All forces were then decomposed along the three directions of the global reference system:

- **Vertical:** axis normal to the gait lab floor, directed upwards,
- **Longitudinal:** axis normal to the vertical one and along the walking direction of the corridor,
- **Lateral:** axis normal to the vertical and longitudinal axis, directed towards the right side.

## 5. Results

The metric used to validate the system is the root mean squared value of the error (*RMSE_j_*) for each Joint Reaction *JR_j_*. In this case, the error is the difference between the Joint Reactions computed starting from the M-Kinect kinematics, $JR_{j}^{M-kinect},$ and those assessed starting from the BTS kinematics, $JR_{j}^{reference}.$ The RMSE_j_ was computed for each joint reaction *j*, adding up all *T_n_* samples of all tests *N*, following Equation (7).

(7) RMSE2j=1N∑n=1…N1Tn∑t=1…Tn(JRjM-kinect(t,n)−JRjreference(t,n))2

Table 3 summarizes the results of all 20 walking tests performed. As can be noticed, the difference between the joint reactions computed using the M-Kinect system and those computed using the BTS reference system is about 1% and the maximum value is 1.5% in case of the vertical right elbow.

**Figure 10:**
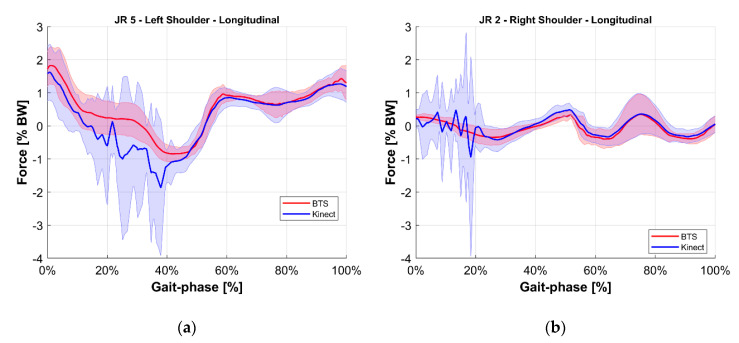
Longitudinal internal reaction force at the shoulder joint. Blue indicates the M-Kinect assessment and red indicates the reference using BTS marker-based mocap. Bold lines represent the average of all tests. The shaded areas represent their standard deviation. (**a**) Left joint and (**b**) right joint. The subject was right-dominant.

**Figure 11:**
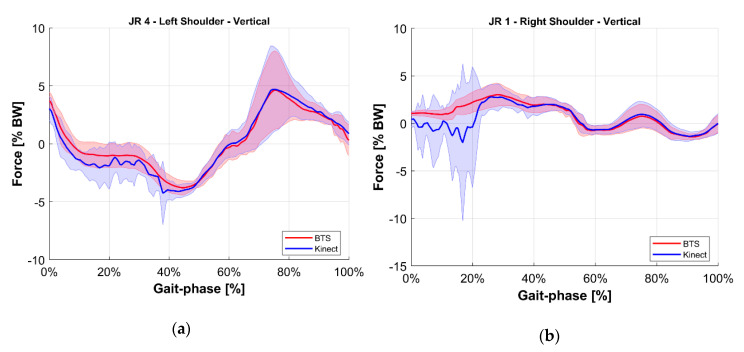
Vertical internal reaction force at the shoulder joint. Blue indicates the M-Kinect assessment. Red indicates the reference using BTS marker-based mocap. Bold lines represent the average of all tests. The shaded areas represent their standard deviation. (**a**) Left joint. (**b**) Right joint. The subject was right-dominant.

**Figure 12:**
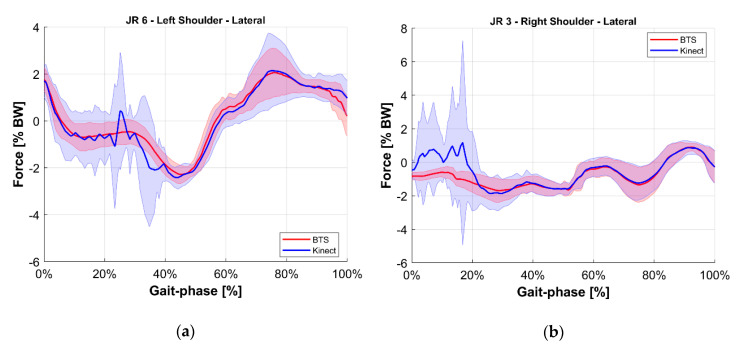
Lateral internal reaction force at the shoulder joint. Blue indicates the M-Kinect assessment. Red indicates the reference using BTS marker-based mocap. Bold lines represent the average of all tests. The shaded areas represent their standard deviation. (**a**) Left joint. (**b**) Right joint. The subject was right-dominant.

**Figure 13:**
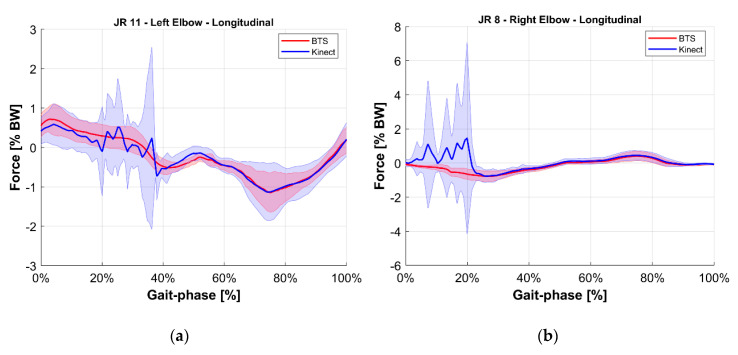
Longitudinal internal reaction force at the elbow joint. Blue indicates the M-Kinect assessment. Red indicates the reference using BTS marker-based mocap. Bold lines represent the average of all tests. The shaded areas represent their standard deviation. (**a**) Left joint. (**b**) Right joint. The subject was right-dominant.

**Figure 14:**
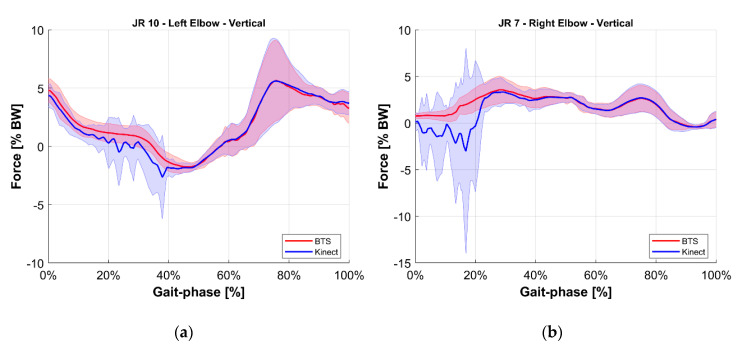
Vertical internal reaction force at the elbow joint. Blue indicates the M-Kinect assessment. Red indicates the reference using BTS marker-based mocap. Bold lines represent the average of all tests. The shaded areas represent their standard deviation. (**a**) Left joint. (**b**) Right joint. The subject was right-dominant.

**Figure 15:**
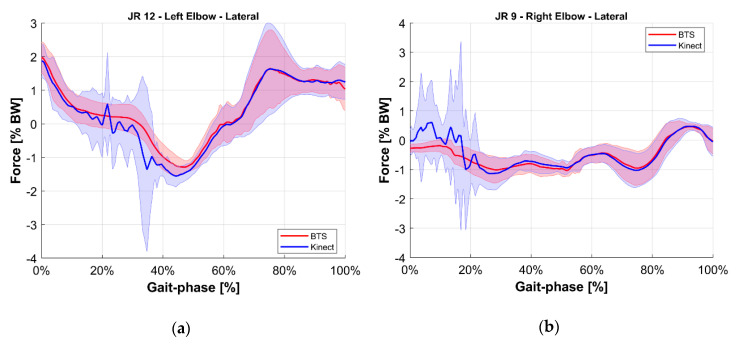
Lateral internal reaction force at the elbow joint. Blue indicates the M-Kinect assessment. Red indicates the reference using BTS marker-based mocap. Bold lines represent the average of all tests. The shaded areas represent their standard deviation. (**a**) Left joint. (**b**) Right joint. The subject was right-dominant.

To ease the comparison, the average value of each joint reaction among all tests at the same gait phase was computed for both the reference BTS system and the M-Kinect system. Results are shown in Figure 10, Figure 11, Figure 12, Figure 13, Figure 14 and Figure 15. To provide a reference of the variability of the forces between different tests, the standard deviation of the assessments among different tests at the same gait phase is shown as a shaded area.

## 6. Discussion

The internal joint reactions, of paramount interest to the therapist providing training, were computed using the M-Kinect data and showed very good correspondence with the ones computed using the reference BTS system data, as shown in Table 3, with a root mean square error closer to the variability of the movement. The proposed data fusion structure, used to create the kinematic data from multiple Kinect units, achieved the desired performance.

However, a minor issue was highlighted from the field test, which was a non-negligible initialization of the fusion process. As can be seen by looking at Figure 13 (right), the estimate from the M-Kinect system is clearly different from the estimate from the reference system in the early phases of gait (0–20%). At the same time, in those phases, the M-Kinect estimate shows high variability. Since this is also the initial part of the recorded kinematic, the issue could be linked with the Kalman filter initialization. The filter initialization takes around 1 s. This time is required to enable the damping of the initial oscillations resulting from the expansion of the kinematic model from a collapsed set of nodes to the skeleton structure. This time can be reduced by setting more constrained values in the initialization of the filter, at the cost of a more unstable response and possibly no convergence of the initialization. Alternatively, the inclusion of more Kinects would provide a wider measurement area, and, thus, the earlier initialization of the model (before the execution of the target gait). This second option implies higher costs, while the hardware and software complexity remain the same given the modular design of the proposed system. A more practical workaround could be a different positioning of the Kinects, which is off-centered with respect to the force platforms, to allow for the filter to settle before the first contact with the force platform, and having the user walk in one direction only.

Another issue could be related to the mechanical model chosen. Different biomechanical models, especially those based on muscular forces, could lead to different solutions. Due to this fact, these results should not be taken as a global reference for joint reaction assessment with other models, but as a tool for relative assessments, e.g., between a pre-training and a post-training behavior. In this case, the simplified rigid-links model was chosen explicitly to allow a robust comparison, which replaced muscular forces with simplified torques, between the kinematic systems.

A limitation of the current study is the involvement of a single expert user as the tester. The system has not been tested with children or non-collaborative users of exoskeletons. The system will be further tested with the same approach involving more users. Many variables could affect walking patterns such as age, gender, size, lesion level, exoskeleton model, and configuration. To provide a comparison focused mainly on the system, this work was limited to an expert user.

## 7. Conclusions

Learning how to use an exoskeleton is not an easy task and teaching a patient how to use it is no less difficult, especially since no quantitative feedback is normally provided. The marker-less motion capture system proposed, in conjunction with instrumented crutches and a simplified mechanical model, provides a low-cost and easy to set-up measuring system, which requires only force platforms on the ground and no instrumentation on the patient himself. This is critical for the application since marker placement on a person not able to stand without robotic support is difficult and time-consuming. The system requires no devices on the exoskeleton or the patient, which simplifies the set-up phase and makes its usage in training, even by non-experts, feasible. The dynamic results show a good reliability of the joints’ internal reaction forces with RMS error values compatible with clinical practice in gait lab assessments.

The total cost of the hardware components of the marker-less motion capture is about 3000 €, which makes the solution affordable in most clinical settings. The instrumented crutches are not commercially available yet even though the hardware is based on off-the-shelf and low-cost components. It is foreseeable that the cost of a pair will be limited to less than 3000 € as well.

Furthermore, future improvements will foresee the reorganization of the system. As for the device considered, Microsoft recently withdrew Kinect from the market. However, the motion capture system in this case presented can be based on almost all the RGB-D sensors such as the Intel RealSense D435. RGB frames can be processed by different skeletonization algorithms. One of the most popular algorithms is Open Pose and can be considered as the state-of-the-art approach for estimating a real-time human pose. Furthermore, a future alternative 3D camera can be represented by the Kinect Azure. Kinect Azure is an innovative and promising development kit equipped with the most advanced sensors of artificial intelligence, which is going to be soon released by Microsoft. A further element that such a change would provide is a sharp separation between the device and the skeletonization processing now performed in the same hardware (closed APIs). No relevant changes are expected in the organization and processing flow. It is worth noting that the minimum number of cameras is four because, to properly obtain the subject skeleton during its walk, it is necessary to register it both from behind and from the front (see Figure 1).

A limitation of the proposed system is the dependence on a set of force platforms, which limits the portability of the solution and increases its cost. A solution, to be investigated in future improvements of the system, may include a mechanical model able to assess the ground reaction forces using the kinematic data integrated with instrumented insoles or with the patient weight data. The accuracy of such a system should be investigated, especially concerning its ability to detect the gait phases [53].

## Figures and Tables

**Table 1:** Nodes and physical markers involved in evaluating distances D1, D2, and D3.

Distances	Nodes	Physical Markers
D1	$x_{p}^{14},\;x_{p}^{19}$	LK, RK
D2	$x_{p}^{19},\;x_{p}^{6}$	RK, LE
D3	$x_{p}^{14},\;x_{p}^{10}$	LK, RE

**Table 2:** Upper joints’ internal reaction forces used as a benchmark.

Variable	Name	Description
JR_1_	Right Shoulder Vertical	Force acting on the torso given by the upper right arm, along the vertical axis of the ground reference.
JR_2_	Right Shoulder Longitudinal	Force acting on the torso given by the upper right arm, along the longitudinal axis of the ground reference.
JR_3_	Right Shoulder Lateral	Force acting on the torso given by the upper right arm, along the lateral axis of the ground reference.
JR_4_	Left Shoulder Vertical	Force acting on the torso given by the upper left arm, along the vertical axis of the ground reference.
JR_5_	Left Shoulder Longitudinal	Force acting on the torso given by the upper left arm, along the longitudinal axis of the ground reference.
JR_6_	Left Shoulder Lateral	Force acting on the torso given by the upper left arm, along the lateral axis of the ground reference.
JR_7_	Right Elbow Vertical	Force acting on the right upper arm given by the right lower arm, along the vertical axis of the ground reference.
JR_8_	Right Elbow Longitudinal	Force acting on the right upper arm given by the right lower arm, along the longitudinal axis of the ground reference.
JR_9_	Right Elbow Lateral	Force acting on the right upper arm given by the right lower arm, along the lateral axis of the ground reference.
JR_10_	Left Elbow Vertical	Force acting on the left upper arm given by the left lower arm, along the vertical axis of the ground reference.
JR_11_	Left Elbow Longitudinal	Force acting on the left upper arm given by the left lower arm, along the longitudinal axis of the ground reference.
JR_12_	Left Elbow Lateral	Force acting on the left upper arm given by the left lower arm, along the lateral axis of the ground reference.

**Table 3:** Comparison between inverse dynamic results. All tests were used. Results are expressed as a percentage of the total weight of subjects and exoskeletons.

Joint Reaction		Root Mean Square Error RMSE (%BW)	
		*Right*	*Left*
Shoulder	Longitudinal	0.4%	0.9%
	Vertical	1.1%	0.9%
	Lateral	0.8%	0.7%
Elbow	Longitudinal	0.8%	0.5%
	Vertical	1.5%	1.0%
	Lateral	0.4%	0.4%

**Figure A1:**
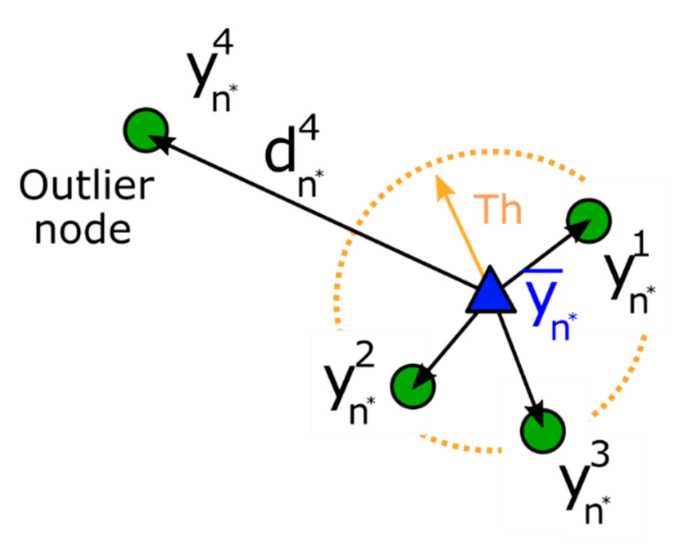
Example of the update procedure for *Sv* with n = n* and M = 4. *Th* indicates the threshold.

## Appendix A

In our system, N = 16 nodes are tracked by M = 4 Kinects, the nth node trajectory (n = 1, …, N), with respect to the global coordinate system that is denoted by $X^{n}={\left[x_{p}^{n},\;x_{v}^{n},\;\;x_{a}^{n}\;\right]}^{T},$ where $x_{P}^{n}={\left[x_{1}^{n},\;x_{2}^{n},\;x_{3}^{n}\;\right]}^{T},$ $x_{v}^{n}={\left[x_{4}^{n},\;x_{5}^{n},\;x_{6}^{n}\;\right]}^{T},$ and $x_{a}^{n}={\left[\;x_{7}^{n},\;x_{8}^{n},\;x_{9}^{n}\;\right]}^{T},$ correspond to the node position, velocity, and acceleration along coordinates x, y, and z, respectively. The underlying mathematical model for the human movement is shown below.

(A1) $$x_{k}=Ax_{k-1}+w_{k}$$

In Equation (A1), the state transition matrix is $A=Diag\left\{A_{1},\cdots ,A_{1}\right\}\in \;{\mathbb{R}}^{9N\times 9N},$ where:

(A2) $$A_{1}=\left[\begin{array}{ccc} I_{3} & I_{3}\Delta t & I_{3}\Delta t^{2}/2\\ 0_{3} & I_{3} & I_{3}\Delta t\\ 0_{3} & 0_{3} & I_{3} \end{array}\right]\in {\mathbb{R}}^{9\times 9}$$

Vector $x_{k}={\left[X^{1},\;\cdots ,\;X^{N}\right]}^{T}\in {\mathbb{R}}^{9N}$ is defined as the 3D joint position, velocity, and acceleration at discrete time k, and Δt is the time between two consecutive sets of data collected from the Kinects. Parameter $w_{k}$ is a random variable representing the process noise. The measurement model for the multi-node tracking is defined below.

(A3) $$j_{k}=Cx_{k}+v_{k}$$

In Equation (A3), vector $y_{k}={\left[Y^{1},\;\cdots ,\;Y^{M}\right]}^{T}\in {\mathbb{R}}^{3NM}$ represents the measurements from the M Kinects at time k. Hence, $Y^{m}={\left[y_{1}^{m},\;\cdots ,\;y_{n}^{m},\;\cdots \;y_{N}^{m}\right]}^{T}$ is the vector of the *N* nodes collected from the *m*th Kinect. Each element $y_{n}^{m}\in {\mathbb{R}}^{3}$ is a vector representing the coordinate along x, y, and z of the nth node measured by the *m*th Kinect at time k. Matrix C $\in {\mathbb{R}}^{3NM\times 9N}$ is the measurement matrix, dynamically built at each *k*th iteration filling a null matrix with a $I_{3\times 3}$ matrix in correspondence of the considered node along columns, and the considered Kinect along the rows.

(A4) $$C=\left[\begin{array}{c} \begin{array}{c} C^{1}\\ \vdots \end{array}\\ \begin{array}{c} C^{m}\\ \vdots \end{array}\\ C^{M} \end{array}\right]$$

where $C^{m}\in {\mathbb{R}}^{3N\times 9N}$ is as follows.

(A5) $$C^{m}=\left[\begin{array}{c} \begin{array}{c} C_{1}^{m}\\ \vdots \end{array}\\ \begin{array}{c} C_{n}^{m}\\ \vdots \end{array}\\ C_{N}^{m} \end{array}\right]$$

In addition, $C_{n}^{m\;}\in {\mathbb{R}}^{3\times 9N}$ is a null matrix with the identity matrix $I_{3\times 3}$ positioned at column 9n + 1. For example, if we consider N = 2 nodes and M = 3 Kinects, the model parameters assume the following form.

(A6) $$x={\left[X^{1},X^{2}\right]}^{T}={\left[x_{p}^{1},\;x_{v}^{1},x_{a}^{1},x_{p}^{2},\;x_{v}^{2},x_{a}^{2}\;\right]}^{T}\;\;\;\;y_{k}={\left[Y^{1},Y^{2},\;Y^{3}\right]}^{T}={\left[y_{1}^{1},y_{2}^{1},y_{1}^{2},y_{2}^{2},y_{1}^{3},y_{2}^{3}\;\right]}^{T}\;\;\;\;C=\left[\begin{array}{cccccc} I_{3\times 3} & 0_{3\times 3} & 0_{3\times 3} & 0_{3\times 3} & 0_{3\times 3} & 0_{3\times 3}\\ 0_{3\times 3} & 0_{3\times 3} & 0_{3\times 3} & I_{3\times 3} & 0_{3\times 3} & 0_{3\times 3}\\ I_{3\times 3} & 0_{3\times 3} & 0_{3\times 3} & 0_{3\times 3} & 0_{3\times 3} & 0_{3\times 3}\\ 0_{3\times 3} & 0_{3\times 3} & 0_{3\times 3} & I_{3\times 3} & 0_{3\times 3} & 0_{3\times 3}\\ I_{3\times 3} & 0_{3\times 3} & 0_{3\times 3} & 0_{3\times 3} & 0_{3\times 3} & 0_{3\times 3}\\ 0_{3\times 3} & 0_{3\times 3} & 0_{3\times 3} & I_{3\times 3} & 0_{3\times 3} & 0_{3\times 3} \end{array}\right]$$

Parameter $v_{k}$ in Equation (A3) is a random variable representing the measurement noise. The process noise and the measurement noise are assumed to be independent of each other with normal probability distributions characterized by the process noise covariance Sw $\in {\mathbb{R}}^{9N\times 9N}$ and measurement noise covariance Sv $\in {\mathbb{R}}^{3NM\times 3NM},$ respectively. The Kalman filter estimates the process state at time k by means of time update equations and obtains feedback in the form of measurements by means of the measurement update equations. In our case, the time update equations assume the following form.

(A7) $${\hat{x}}_{k}^{-}=A\cdot {\hat{x}}_{k-1}\;\;\;\;P_{k}^{-}=A\cdot P_{k-1}\cdot A^{T}+Sw\;\;\;$$

In Equation (A7), ${\hat{x}}_{k}^{-}\;$is the a priori state estimate at step k, obtained from the process state estimate calculated at step *k-1*, ${\hat{x}}_{k-1}$. $P_{k}^{-}$ $\in {\mathbb{R}}^{9N\times 9N}$ is the a priori estimate error covariance. $P_{k-1}$ $\in {\mathbb{R}}^{9N\times 9N}$ is the a posteriori estimate error covariance at *k − 1*. Matrix $Sw=Diag\left\{Sw_{1},\cdots ,Sw_{n}\;\cdots ,Sw_{N}\right\}\in \;{\mathbb{R}}^{9N\times 9N}$, where $Sw_{n}\in {\mathbb{R}}^{9\times 9}$.

(A8) $$Sw_{n}=\left[\begin{array}{ccc} I_{3}\Delta t^{4}/4 & I_{3}\Delta t^{3}/2 & I_{3}\Delta t^{2}/2\\ I_{3}\Delta t^{3}/2 & I_{3}\Delta t^{2} & I_{3}\Delta t\\ I_{3}\Delta t^{2}/2 & I_{3}\Delta t & I_{3} \end{array}\right]\cdot Sw_{i}$$

In Equation (A8), parameter $Sw_{i}$ is fixed and set to the value $Sw_{i}=0.05^{2}$ (m/s^2^)^2^ [66,75].

The state error covariance matrix is $P=Diag\left\{P_{1},\;\cdots ,P_{n},\cdots ,P_{N}\right\}$ with sub-matrix $P_{n}=\left[\begin{array}{ccc} \sigma_{p}^{2} & 0_{3\times 3} & 0_{3\times 3}\\ 0_{3\times 3} & \sigma_{v}^{2} & 0_{3\times 3}\\ 0_{3\times 3} & 0_{3\times 3} & \sigma_{a}^{2} \end{array}\right]{\mathbb{R}}^{9N\times 9N}$ and parameters $\sigma_{p}^{2},\;\sigma_{v}^{2},$ and $\sigma_{a}^{2}$ are the variances of position, velocity, and acceleration of the state.

The measurement update equations are shown below.

(A9) $$K_{k}=P_{k}^{-}\cdot C^{T}\cdot {\left(C\cdot P_{k}^{-}\cdot C^{T}+Sv_{k}\right)}^{-1}\;\;\;\;{\hat{x}}_{k}={\hat{x}}_{k}^{-}+K_{k}\cdot \left(y_{k}-C\cdot {\hat{x}}_{k}^{-}\right)\;\;\;\;P_{k}=\left(I-K_{k}\cdot C\right)\cdot P_{k}^{-}\;\;\;$$

In Equation (A9), $K_{k}$ is the Kalman gain, a matrix ${\mathbb{R}}^{9N\times 3NM},$ acting as the blending factor that minimizes the a posteriori error covariance $P_{k}.$ Matrix $Sv=Diag\left\{Sv^{1},\cdots ,Sv^{m},\;\cdots ,Sv^{M}\right\}$ with sub-matrix $Sv^{m}=Diag\left\{Sv_{1}^{m},\cdots ,Sv_{n}^{m},\;\cdots ,Sv_{N}^{m}\right\},$ and $Sv_{n}^{m}=\left[I_{3\times 3}\cdot \sigma_{Y}^{2}\right]\in \;{\mathbb{R}}^{3\times 3},$ expresses the standard uncertainty associated to each $y_{n}^{m}$ measurement. The filter is initialized as follows: ${\hat{x}}_{0}=C^{T}\cdot y_{0}$ where $y_{0}$ is the first set of acquired nodes. Matrix Sw is initialized using $Sw_{i}=0.05^{2}$ (m/s^2^)^2^ and the matrix *P* is initially set equal to Sw. The matrix Sv is initialized using $\sigma_{Y}^{2}=0.005^{2}$ m^2^.

At each *k*th step, ${\hat{x}}_{k}^{-}$ and $P_{k}^{-}$ are estimated. The Kalman gain is calculated and used to estimate the current state ${\hat{x}}_{k}$ and its associated error covariance $P_{k}.$

The measurement noise covariance matrix Sv is updated at every time instant *k* using a dedicated procedure to minimize the influence of outlier nodes. Figure A1 explains the situation for n = n^*^ and M = 4. In this case, $y_{n^{*}}^{4}$ is the outlier node, which decreases the influence of its measurement. We performed the following steps.

1. We calculate the median value among the four measurements, ${\bar{y}}_{n^{*}}=median\;\left\{y_{n^{*}}^{1},y_{n^{*}}^{m},y_{n^{*}}^{M}\right\}$;
2. For each of the m nodes, the distance $d_{n^{*}}^{m}$ from ${\bar{y}}_{n^{*}}$ is computed, $d_{n^{*}}^{m}=\left|y_{n^{*}}^{m}-{\bar{y}}_{n^{*}}\right|$;
3. The matrix Sv is updated by considering a modified value for $\sigma_{Y}^{2}$ in each node by applying the notation reported in Equation (A10).
  (A10) $$\sigma_{Y^{m}.n^{*}}^{2}=\{\begin{array}{cc} \sigma_{Y}^{2} & d_{n^{*}}^{m}\leq Th\\ \frac{d_{n^{*}}^{m}}{Th}\sigma_{Y}^{2} & d_{n^{*}}^{m}>Th \end{array}$$

In this case, Th represents a threshold value set to 1 cm. A $d_{n^{*}}^{m}$ value higher than Th causes a linear amplification of the measurement uncertainty for the *m*th node, and, thus, its lower influence in the Kalman filter update.

Measurements $y_{k}$ undergo a suitably developed pre-processing called Orientation Testing before being used in Equation (A9). The Orientation Testing has been developed to consider the fact that some Kinects will see the subject from behind, and that no prior information is available to define which devices or subjects will be affected by this. Therefore, some measurements $Y^{m}$ undergo a mirroring effect. In this case, the measurements $y_{9}^{m},y_{10}^{m},y_{11}^{m},y_{17}^{m},y_{18}^{m},\;\mathrm{and}\;y_{19}^{m}\;$of the right limbs, and the measurements $y_{5}^{m},y_{6}^{m},y_{7}^{m},y_{13}^{m},y_{14}^{m},\;\mathrm{and}\;y_{15}^{m}\;$of the left limbs are associated to positions $x_{X}^{5},x_{X}^{6},x_{X}^{7},x_{X}^{13},x_{X}^{14},\;\mathrm{and}\;x_{X}^{15}$ and $x_{X}^{9},x_{X}^{10},x_{X}^{11},x_{X}^{17},x_{X}^{18},\;\mathrm{and}\;x_{X}^{19}$ of the a priori state estimate respectively, which yields to an erroneous estimate of the state ${\hat{x}}_{k}.$ To avoid this, at each iteration k, the following quantities are computed.

(A11) $$D_{f}=\underset{r}{\sum }\left|{\hat{x}}_{X}^{-r}-y_{r}^{m}\right|+\underset{l}{\sum }\left|{\hat{x}}_{X}^{-l}-y_{l}^{m}\right|\;\;\;\;D_{b}=\underset{r}{\sum }\left|{\hat{x}}_{X}^{-r}-y_{l}^{m}\right|+\underset{l}{\sum }\left|{\hat{x}}_{X}^{-l}-y_{r}^{m}\right|\;\;\;$$

In these expressions, $D_{f}$ and $D_{b}$ are cumulative distances computed by considering both the possibilities, i.e., that the Kinect is placed in front of ($D_{f}$) and behind ($D_{b}$) the body. In the first case, it will be $D_{f}<D_{b},$ and the measurement assignment is left unchanged. In the second case, it will be $D_{f}>D_{b},$ and the measurements are assigned to the opposite side.
